# Supplementary material for: Complementary Transcriptomic and Proteomic Analysis Reveals a Complex Network Regulating Pollen Abortion in GMS (msc-1) Pepper (Capsicum annuum L.)
Source: Int J Mol Sci. 2019 Apr 11;20(7):1789. doi: 10.3390/ijms20071789 (PMC6480423; doi:10.3390/ijms20071789)
Supplement: Supplementary file 1 [file ijms-20-01789-s001.zip › Supplementary.pdf]

Supplementary materials

Table S1 Overview of protein identification

Order number	Total spectra	Spectra	Peptide	Unique peptide	Protein
16C1369A-1	82970	25183	20844	18348	2769
16C1369A-2	83893	24955	20653	18181	2823
16C1369A-3	82005	24350	20311	17893	3289
16C1369B-1	84636	25352	20306	17907	2571
16C1369B-2	84668	25391	20279	17848	2587
16C1369B-3	84044	25621	20440	18006	2756
Total	502216	150852	29056	26108	4909

Table S3 Summary of RNA sequencing and assembly

Sample Name	Clean Reads	Genome Map Rate	Unique genes
16C1369A-1	47179448	89.07%	25168
16C1369A-2	39567516	88.84%	24941
16C1369A-3	45583918	88.47%	25035
16C1369B-1	46992664	87.75%	25454
16C1369B-2	43014098	87.38%	25176
16C1369B-3	41683754	88.76%	25221

Table S10. Primers used in this study.

Gene name	Gene ID	Forward sequence (5'→3')	Reverse sequence (5'→3')
**ACOS5**	Capana02g003302	CCAGCTGAATTAGAGGGGATCC	CTCGTTTATAATGTGCAGCGGT
**PKSA**	Capana01g003460	GGTGATCAACTGAAGGAGACCA	CCCCATTCTTCACCACCATCTT
**PKSB**	Capana08g002676	GGGCAGTTCATCCAGGTGG	TGTGATTCCAGGACCAAATGCA
**MS2**	Capana03g003125	AGGAAGTCAGTGGAGCAAGC	CCTTAGCCCTGGAATGTGGA
**TKPR1**	Capana05g000665	ACCAACTTGCCTCACTTCTATCA	TCGTCAAACATCTCTTCAATGGAC
**TKPR1**	Capana01g002831	ACAGAAGGTTGGGGTTTGTTCA	CTATGTGCCATGTCTTCCCCTT
**CYP704B**	Capana01g002203	GAATACAATTGGGGCTCTGATGC	CCCAGGCACCAACTTGAATTTAT
**ABCG26**	Capana07g002406	AAGGGCTGGAATGGTTGCTT	ACCAACAGTGTCAAATGAAGGTG
**AMS**	Capana08g000254	AGACTGGTGGATTTGTGAGGATC	CTAGCCATTTCAGACCAACCTCT
**TDF1**	Capana04g001901	AGTACACAGCTACTCCCCTTCT	GGCTGCTTTTCCTCAACTTCAG
**ATXR6**	Capana03g001971	CCGTGAAGCTGATGATGGAGAT	CTCGGCTTTCACCATTAACGTC
**A7**	Capana07g001721	AGAAACTCACACGCGAATGTTG	GCGTTCAGAGATGGGGAAACTA
**ACA9**	Capana03g000026	CCCCTCTTCTCTGGTGTTATAGG	GGTGTCTTTGGAACTGGGATGA
**4CL3**	Capana03g001733	CAAAAGGATGATGCTGCAGGG	TGGCTCTCAGTTCTTTCCTCAG
**GATL4**	Capana01g003063	TTTGCAGGAAACATCAAGGCAG	TAACGCAACTCTGGATGAACGA
**ARO1**	Capana08g002699	CCAAGCACCTAGTACCACATGT	CGTGAGCCTCTGGACTGATAAA
**AGL62**	Trans_newGene_14655	AGCGGAGAAGAAGAAGAAAGAGA	GATCCTCAGCTCCAAATCTTGC
**FAS1**	Capana06g000774	TGGCATTGATCAAGTCCTGGAA	ATGAGCAGCAGATTTCAGTGGT
**LAP3**	Capana03g003177	TACCTGGCTAGATTGATGGGGA	ATGGAAGGGTGGCAATATGGTT
**PG2**	Capana11g001305	AATGTCATTGTTCGAACCACCG	TATCAGCTTCATAGACCCGCAC
**TA29**	Capana02g001969	ATGTCCACGCAACAGTCCAT	CTTCCTCTCGCTGCTCTGAC
**TA1**	Capana04g001989	AGTTGGGCATTCACGGGATT	TGGCCAGTCACAAAACCAGA
**UBI-3**	AY486137.1	CACATGTACAAGACTGACAGGGCCA	AGACCCGTTCCTTGACAACCCAC

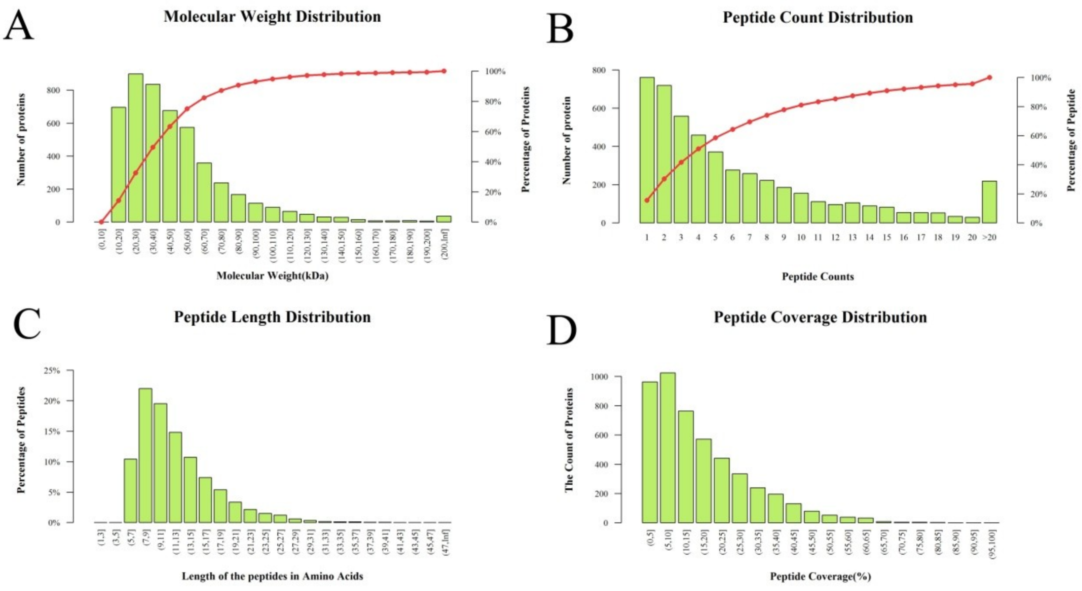

Figure S1. Summary of the label-free proteome. A: Molecular weights distribution of identified proteins; B: Number of peptides that match to unique proteins; C: Length distribution of identified peptides; D: Distribution of coverage of the proteins by the identified peptides.

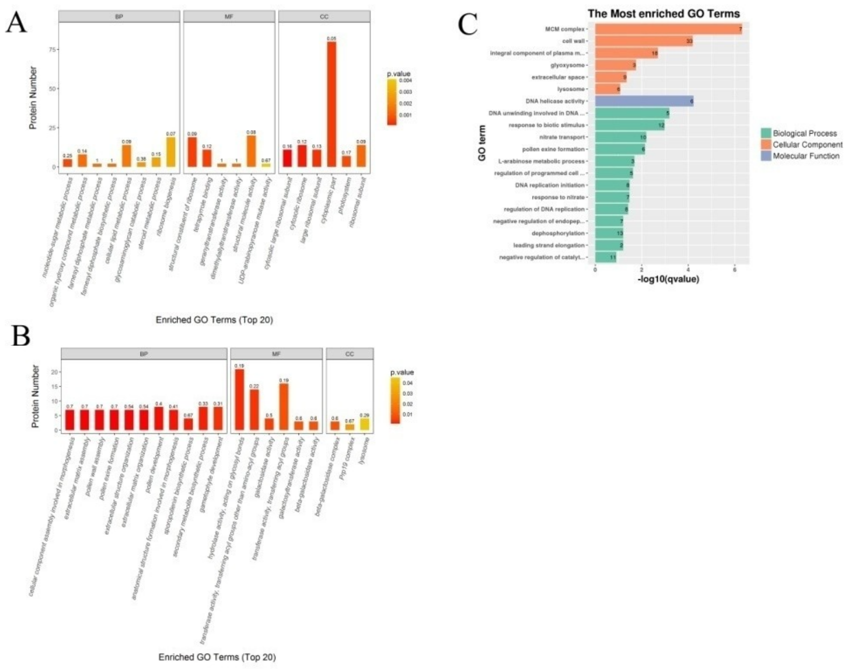

Figure S2. GO functional classification based on DEPs and DEGs. A:GO functional classification based on the 182 DEPs in the proteome; B:GO functional classification based onthe 492 DEPs (presence/absence proteins) in the proteome; C: GO functional classification based on the 1069 DEGs in the transcriptome.

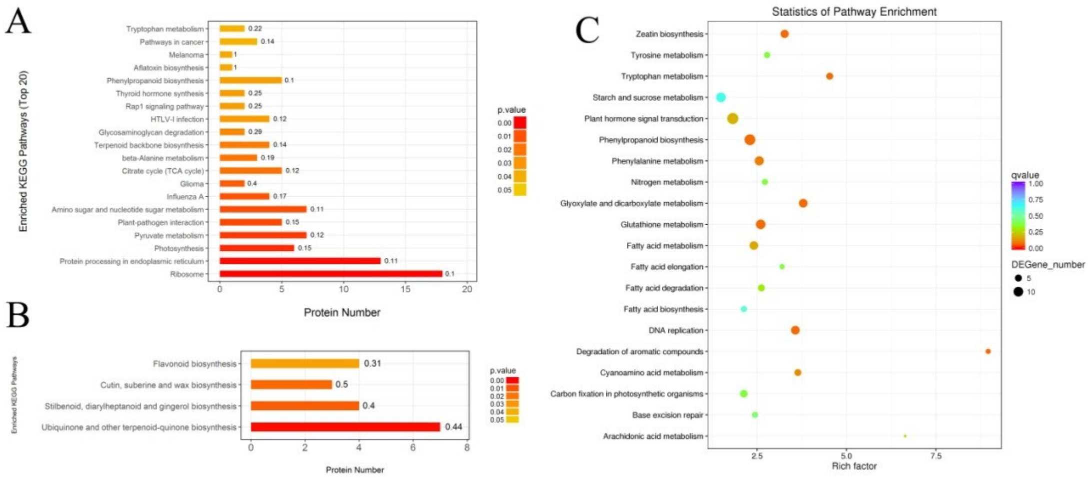

Figure S3. KEGG pathways associated with DEPs and DEGs. A: KEGG pathways associated withthe 182 DEPs in the proteome; B: KEGG pathways associated with the 492 DEPs (presence/absence proteins) in the proteome; C: KEGG pathways associated with the 1069 DEGs in the transcriptome.
