# Supplementary figures and images for: Complementary Transcriptomic and Proteomic Analysis Reveals a Complex Network Regulating Pollen Abortion in GMS (msc-1) Pepper (Capsicum annuum L.)
Source: Int J Mol Sci. 2019 Apr 11;20(7):1789. doi: 10.3390/ijms20071789 (PMC6480423; doi:10.3390/ijms20071789)

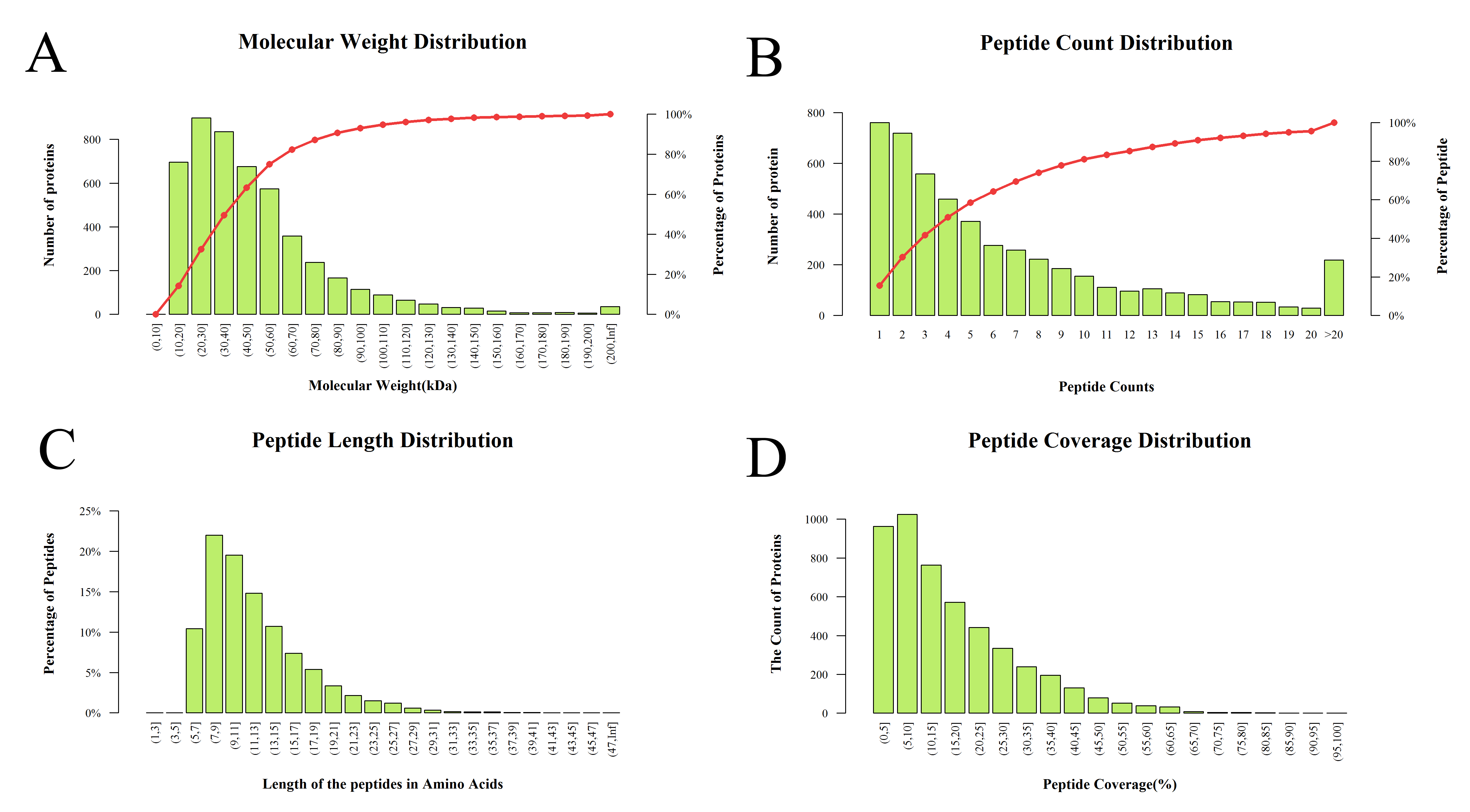

Supplement: Supplementary file 1 [file ijms-20-01789-s001.zip › Figure S1.tif]

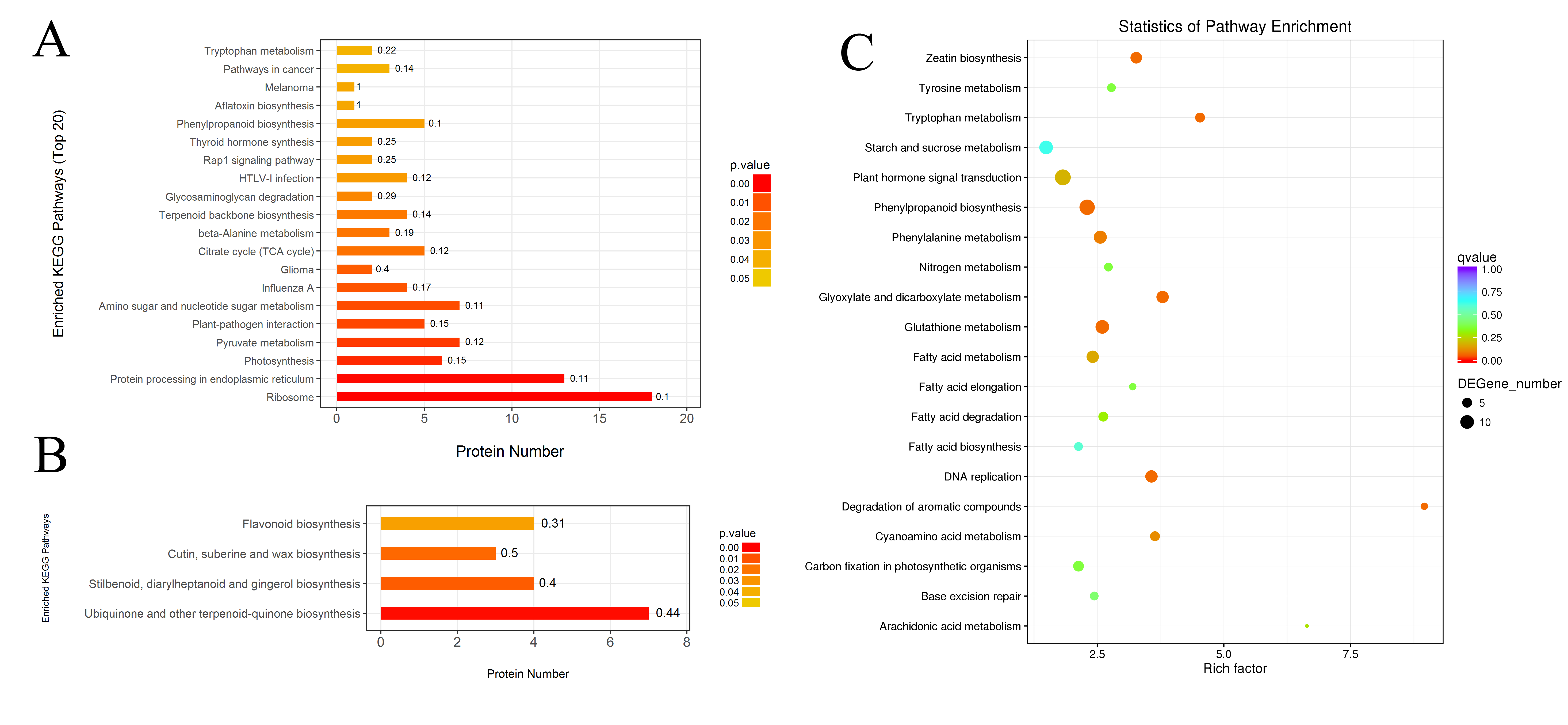

Supplement: Supplementary file 1 [file ijms-20-01789-s001.zip › Figure S3.tif]
